# Study on the Linezolid Prescription According to the Approval of Indication in a University Hospital

**Published:** 2015

**Authors:** Manuela Pérez-Cebrián, María M. Morales Suárez-Varela, Isabel Font-Noguera, Emilio Monte-Boquet, Jose Luís Poveda-Andrés, Jose María Martín-Moreno, Nuria Rubio-López, Elias Ruiz-Rojo, Agustín Llopis-González

**Affiliations:** a *Pharmacy Service. University Hospital La Fe, Valencia, Spain.*; b *Unit of Public Health and Environmental Care, Department of Preventive Medicine, University of Valencia, Spain.*; c *CIBER of Epidemiology and Public Health (CIBERESP), Madrid, Spain. *; d *Center for Public Health Research (CSISP), Valencia, Spain.*; e *Conselleria de Sanidad, Valencia, Spain.*

**Keywords:** Approval of indications, Drug-related problems, Drug safety evaluation, Linezolid prescription, University hospital

## Abstract

Indications for linezolid use are nosocomial or community-acquired pneumonia and skin infections or soft tissue infection caused by gram-positive microorganisms, but new recommendations may emerge. It is important to balance benefits with risks because severe adverse events have been described in patients taking linezolid treatment. Accordingly, we evaluated the suitability of linezolid prescription according to approval of indication by evaluating the presence of drug-related problems (DRP) in a University hospital. DRP were identified in 36 patients (50.0%). In most cases, they were related to known or established indications (15 patients, 20.8%), to safety (5 patients, 6.9%), and to both in others (16 patients, 22.2%). No DRP were recorded, which modified linezolid efficacy. DRP were significantly higher in the patients treated by an approved indication in Spain (63.3%) than in those treated by an unapproved indication in Spain (28.6%). We concluded that new studies about extending linezolid indications may be necessary.

## Introduction

Linezolid is the first compound belonging to a new class of antibiotics: oxazolidinones. Unlike other antibiotics, oxazolidinones work by inhibiting the synthesis of bacterial proteins in an early phase more quickly than other antibacterial agents. Specifically, it joins itself to the bacterial ribosome and prevents from the formation of functional initiation complex 70S, which is essential for translation (1,2). Since its approval by the European Agency for the Evaluation of Medicinal Products (EMEA) in 2001, linezolid has been included in the therapeutic arsenal as an active agent against Staphylococcus and multi-resistant bacteria (3). In 2007, the Spanish Agency for the Evaluation of Medicinal and Health Products (AEMPS) (4), in coordination with other European Health Authorities, approved the indication of linazolid use in Spain for complicated skin and soft tissue infections, and also for nosocomial or community-acquired pneumonia but disapproved it for other indications. Recent findings have identified the possible utility of linazolid as a suitable candidate to treat other infections (5, 6).

Initially, the indications considered for its use were nosocomial or community-acquired pneumonia due to degree of penetration in pulmonary tissue (7), and infections of the skin or soft tissue arising from gram-positive microorganisms, which are susceptible to this antibiotic. Despite the promising clinical and experimental data, there are still questions pending on issues such as effectiveness, and the possibility of association with efficacy and toxicity in prolonged treatment. This is particularly true for patients with severe infections who are hospitalized in resuscitation units (8). For this reason, it was recommended to restrict the use of linezolid to infections caused by multi-resistant gram-positive microorganisms.

The standard therapy for methicillin-resistant Staphylococcus aureus (MRSA) has been traditionally based on vancomycin administration. However, linezolid is theoretically more effective to treat MRSA because it is available intravenously and orally, thus theoretically promoting reduced hospital stays (9). Others argue that with appropriately used antibiotics, new recommendations for linezolid prescription to treat patients may emerge (10).

In order to assess Linazolid, we can employ drug-related problems (DRP), which are an event or circumstance involving drug therapy, which actually or potentially interferes with desired health outcomes.

The aim of this study was to evaluate the suitability of linezolid prescription according to approval of indication by evaluating the presence of Drug-Related Problems (DRP) in a University hospital in Spain. 

## Experimental

*Materials and methods*

We carried out an observational prospective study with a cohort of patients in a noncritical condition who were hospitalized in the largest university-teaching hospital in Valencia, Spain (the La Fe University Hospital), with 1,500 beds, between July 2007 and January 2008, following the only condition of having begun intravenous or oral linezolid treatment. Excluded subjects were: pediatric patients (<18 years old); patients admitted to haematology, oncology, resuscitation and intensive care; patients seen for a period less than 24 h; patients transferred to resuscitation or intensive care units after beginning treatment; and patients re-admitted within 7 days of discharge. Finally, we identified 72 patients.

Each patient was registered on a tracking sheet to monitor clinical development and to record the date when the treatment was finished. 

While the patients taking linezolid, they were monitored throughout the treatment period, further variable data was collected, as so:

- demography (age, sex)
- admission, including the prescribing linezolid department and average duration of stay.
- diagnosis, indicating if there was approved indication in Spain (AIS) or an unapproved indication in Spain (UIS) by the Spanish Agency of Medication and Health Products (SMHPA).
- Treatment characteristics, including antibiogram, sensitivity to linezolid, concomitant antibiotics and treatment duration.

Moreover, those situations which could cause real or potential DRP as regards indications and safety were also recorded. 

A drug therapy- (related) problem (DTP) can be defined as an event or circumstance involving drug treatment which actually or potentially interferes with the patient experiencing optimum outcome of medical care. In 1990, L.M. (11). Strand and colleagues classified DTP into different categories. According to these categories, pharmacists generated a list of DTP for each patient. Consequently, pharmacists obtained a clearer picture of the patient's drug therapy and medical conditions.

We stratified both the clinical diagnoses and the DTR identified after treatment with linazolid (Tables 4 and 5) in both AIS and UIS in order to compare them.

All the parameters corresponding to the above-defined variables were recorded in an Access database (Microsoft Office 2007), which was specifically designed for this purpose. The SPSS PC+ (version 17.0) statistical package was used for statistical processing. 

The statistical analysis included the descriptive statistics used to compare proportions (Chi-square test). A level of p≤0.05 in the bilateral comparison was considered statistically significant. 

## Results

During the study period, 5,109 admissions were recorded in the hospital departments prescribing linezolid. In all these departments, 75 patients (1.5% of total admissions) were treated with linezolid, of whom 3 were excluded: one of them due to short treatment duration (< 24 h); and two others due to readmission within 7 days of the previous episode. Therefore, 72 linezolid-treated patients participated in this study (63.9% men). The mean age of the participants was 54.0±19.1 years. Of the 72 study patients, 31 (43.1%) were administered linezolid exclusively by intravenous administration, 20 (27.8%) by oral administration, and 21 (29.2%) both intravenously and orally. Mean linezolid treatment duration was 16.2±17.5 days. Ten patients were treated for more than 28 days (maximum recommended treatment duration). In 24 patients (33.3%), the only anti-infectious treatment used was linezolid. In 48 (66.6%), linezolid treatment was concomitantly administered with another antibiotic, while a third antimicrobial agent was associated in 13 patients (18.1%); the most commonly used concomitant antibiotic was imipenem/cilastatine; 12.5%, followed by levofloxacin in 7 (9.7%). Finally, 14 patients presented moderate renal insufficiency upon admission (plasmatic level of creatinine, >1.4 mg/dl) (Table 1).

**Table 1:** Treatment information and demographic and clinical characteristics (N=72).

**Variable**	**Mean**	**SD**
Age (years)	54.0	19.1
Length of linezolid treatment (days)	16.2	17.5
	N (%)	95% CI
Men	46 (63.9)	52.3-74.3
Type of administration		
Intravenously	31 (43.1)	32.0-54.7
Orally	20 (27.8)	18.4-38.9
Intravenously and orally	21 (29.2)	19.6-40.4
Concomitant anti-infectious treatments	48 (66.6)	55.2-76.8
Renal insufficiency at the time of admission	14 (19.4)	11.5-29.8

SD, Standard Deviation

Renal insufficiency, plasmatic creatinine level >1.4 mg/dl.

Table 2 shows the distribution of the study patients into the prescribing linezolid hospital departments. We observe that the main sources of patients were the departments of Nephrology, Pneumology and Neurosurgery (9 patients each, 12.5%), followed by the departments of Infectious Diseases (7 patients, 9.7%) and Thoracic Surgery and Lung Transplants (6 patients each, 8.3%). 

**Table 2:** Distribution of the study patients into the prescribing linezolid departments of the hospital

**Prescribing linezolid departments**	**N (%)**	**95% CI**
Nephrology	9 (12.5)	6.3-21.7
Pneumology	9 (12.5)	6.3-21.7
Neurosurgery	9 (12.5)	6.3-21.7
Infectious diseases	7 (9.7)	4.3-18.3
Lung transplant unit	6 (8.3)	3.4-16.5
Thoracic surgery	6 (8.3)	3.4-16.5
Internal medicine	6 (8.3)	3.4-16.5
General surgery	5 (6.9)	2.6-14.7
Vascular surgery	4 (5.6)	1.8-12.9
Hepatology	4 (5.6)	1.8-12.9
Cardiac surgery	3 (4.2)	1.1-10.9
Digestive medicine	2 (2.8)	0.5-8.9
Urology	1 (1.4)	0.1-6.7
Cardiology	1 (1.4)	0.1-6.7
Total	72 (100.0)	95.9-100.0

95% CI, 95% Confidence Interval.

Table 3 shows the diagnoses of the patients treated with linezolid depending on whether there was an AIS or an UIS. We observe that the most common linezolid indication among the study patients was pneumonia (12 patients, 16.7%), followed by infections from surgical wounds and cystic fibrosis (8 patients each, 11.1%). These indications are all AIS. We found that 44 patients (61.1%) were treated by an AIS, while 28 (38.9%) were treated by an UIS.

**Table 3:** Clinical diagnoses of patients treated with linezolid according to type of indication (approved in Spain (AIS) or unapproved in Spain (UIS)).

**Clinical diagnosis**	**N**	**95% CI**
AIS		
Pneumonia	12 (16.6)	9.4-26.6
Cystic fibrosis/ pneumonia	8 (11.1)	5.3-20.0
Infections of the skin or soft tissue	-	-
Infection of surgical wound	8 (11.1)	5.3-20.0
Ischemia MMII degree IV	4 (5.6)	1.8-12.9
Cutaneous ulcer	1 (1.4)	0.1-6.7
Open trauma	3 (4.1)	1.1-10.9
Costal wall cellulitis	1 (1.4)	0.1-6.7
Lower limb cellulitis	4 (5.6)	1.8-12.9
Abscess	3 (4.2)	1.1-10.9
Total	44 (61.1)	49.5-71.8
UIS		
Aneurism	5 (6.9)	2.6-14.7
Streptococcal endocarditis	1 (1.4)	0.1-6.7
Inflammatory intestinal disease	2 (2.8)	0.5-8.9
Aortic stenosis	1 (1.4)	0.1-6.7
Hepatic failure	2 (2.8)	0.5-8.9
Glomerular nephritis	2 (2.8)	0.5-8.9
Neoplasia	4 (5.6)	1.8-12.9
Osteomyelitis	1 (1.4)	0.1-6.7
Peritonitis	1 (1.4)	0.1-6.7
Sepsis	5 (6.9)	2.6-14.7
Catheter dialysis infection	3 (4.1)	1.1-10.9
Spondylitis	1 (1.4)	0.1-6.7
Total	28 (38.9)	28.2-50.5

95% CI, 95% Confidence Interval.

Table 4 indicates the microorganisms identified in the study patients according to their sensitivity to linezolid. Microbiological cultures were obtained from 66 patients (91.7%), and the result was negative for 6 of them (9.1%). In 40 of the 60 patients who gave a positive result, the antibiogram showed sensitivity to linezolid. Of these 40 patients, the most commonly identified microorganism was coagulase-negative staphylococcus (14 patients, 35.0%). 

**Table 4:** Microorganisms identified in the study patients according to sensitivity to linezolid

	**N (%)**	**95% CI**
Absence of culture	6 (8.3)	3.4-16.5
Obtaining culture	66 (91.7)	83.5-96.5
Negative culture	6 (9.1)	3.8-17.9
Positive culture	60 (90.9)	82.0-96.2
Microorganism with no proven sensitivity to linezolid	20 (33.3)	22.3-45.9
Microorganism with proven sensitivity to linezolid	40 (66.7)	54.1-77.7
Aspergillus versicolor	1 (2.5)	0.1-11.7
Chryseobacterium meningospticum	1 (2.5)	0.1-11.7
Corynebacterium sp.	1 (2.5)	0.1-11.7
Enterococcus faecalis	1 (2.5)	0.1-11.7
Enterococcus gallinarum	1 (2.5)	0.1-11.7
Enterococcus sp.	1 (2.5)	0.1-11.7
Prenotella loescheii	1 (2.5)	0.1-11.7
Staphylococcus aureus	7 (17.5)	8.0-31.6
Staphylococcus aureus (methicillin-**resistant**)	11 (27.5)	15.4-42.8
Staphyloccus coagulase negative	14 (35.0)	21.5-50.6
Staphyloccus homini	1 (2.5)	0.1-11.7
Total	40 (100.0)	92.8-100.0

95% CI, 95% Confidence Interval.

Altogether, DRP related to linezolid were detected in 36 patients (50.0%). These DRP were all significantly higher in the patients treated with linezolid by an AIS (63.6%) than in those treated by an UIS (28.6%); p-value=0.004.

Of the 36 DRP related to linezolid detected, 15 (41.7%) referred to known or established indications, 5 (13.9%) to safety, and 16 (44.4%) to both indications and safety. No differences were found between patients treated with linezolid by an AIS and those by an UIS (Table 5).

**Table 5:** Drug-related problems (DRP) according to the type of indication of linezolid (approved in Spain (AIS) or unapproved indication in Spain (UIS)).

**DRP type**	AIS (N=44)		UIS (N=28)		**p-value**
	**N (%)**	**CI 95%**	**N (%)**	**CI 95%**	
Indication	12 (27.3)	15.7-41.7	3 (10.7)	2.8-26.4	0.092
Safety	4 (9.1)	3.0-20.5	1 (3.6)	0.2-16.4	0.672
Indication + Safety	12 (27.3)	15.7-41.7	4 (14.3)	4.7-31.0	0.196
Total	28 (63.6)	48.7-76.8	8 (28.6)	14.2-47.1	0.004

CI 95%, 95% Confidence Interval

p-value, Chi-square test

## Discussion

In the present study, we found that the DRP in the use of Linazolid in Spain were identified in 50% of the study patients. In most cases, they were related to indications (20.8%), to safety in others (6.9%) and even to both (22.2%). No DRP were recorded, which modified linezolid efficacy. DRP were significantly higher in the patients treated by AIS (63.3%) than those treated by an UIS (28.6%).

Pharmacological monitoring, classified by clinical area, permitted us to study 72 patients treated with linezolid; that is, 1.5% of total admissions in the hospital departments prescribing linezolid over a 7-month period. This use seems quite widespread when considering the restricted nature of the drug, and it could be due to a possible cause inferred from the study results; that is, there is often a high infection rate due to gram-positive multi-resistant microorganisms, which is a pressing problem in numerous hospitals. 

As previously stated, linezolid is one of the main alternatives to vancomycin to treat infections caused by MRSA. However, this was the indication (using strict criteria with culture and antibiogram documentation) in only 15.3% of the patients. The overall infection rate due to MRSA in the study patients admitted to the hospital departments was 2.1%, which is slightly higher than the 1.5% rate observed in all the hospital departments over the same period. This is because the study included the units which traditionally present high MRSA prevalence. The main source of patients came from the Pneumology ward, with a high proportion of patients with pneumonia and cystic fibrosis, followed by the Nephrology ward, where catheter manipulation favours the colonisation of gram-positive microorganisms. 

The exclusion criteria for the study patients were based on a former bibliographic review which focused on avoiding information biases in the results.

One of the principal strategies to control MRSA from propagating in the community is based on the detection of possible carriers, hygienic measures, and the isolation of the colonised or infected patients (12). After their recent extensive bibliographic review, Avdic and Cosgrove (13) proposed emphasizing the importance of opening and draining purulent lesions and of attending wounds; adjuvant antibiotic treatment should be specified according to the localization and extension of the disease, the systemic symptoms and the risk factors noted in each patient. The best treatment for this pathogen has not yet been determined, except for the use of non-beta-lactamase antibiotics, such as trimetoprim/sulphametoxazol, clindamicine, tetracycline and linezolid. Vancomycin and daptomicine should also be considered a parenteral therapy and severe pathologies (pneumonia or necrotic fasciitis) may require being admitted into an ICU (13, 14). 

Although vancomycin-resistant enterococci (VRE) prevalence is low (1-4%) in Spain, its rise can be attributed to the extended use of vancomycin (15). Vancomycin continues to be a gold-standard option for the treatment of MRSA, although linezolid, minocycline, daptomycine and tigecycline are considered more effective as they also avoid increased resistance to staphylococci and VRE prevalence (16). Thus, linezolid is proposed as an alternative to vancomycin to treat MRSA in nosocomial pneumonia, especially in patients with renal failure, for whom vancomycin (which obeys a concentration-dependent kinetics and whose dosage should be based on creatinine clearance) is frequently underdosed (17).

One of the main purposes of the protocols and guidelines to promote the rational use of antibiotics is the precise compliance with their indications. In 2007, the EMEA approved the use of linezolid to treat community-acquired and nosocomial pneumonia, as well as infections of the skin and the soft tissue resulting from gram-positive microorganisms. Bacteraemia is not mentioned in the therapeutics indications section of the EU label. The US label contains a more extensive list of therapeutic indications, which includes uncomplicated skin and skins structures infections, and the description of specific pathogens for each indication. The absence of pathogenic germ specification, combined with different diagnoses, can cause variation in prescriptions, and even distinct interpretations (18). 

In the present study, the indications in 28 cases (38.9%) do not correspond to those approved because of the vast variation in the pathological processes motivating its use. However, this should be considered with much caution as diagnosis upon admission does not necessarily reflect the ensuing septic complications which might have motivated linezolid prescription.

Given the variety of samples and isolated organisms, concomitant antibiotic treatment was used in 66.6% of the patients, and the most common of these was imipenem/cilastatin and levofloxacin. These antibiotics duplicate the effect of linezolid by covering a similar or extended spectrum of activity via different mechanisms. In other cases, additional antibiotics were needed given the confirmed sensitivities.

Although creatinine clearance was not specifically determined in our patients, a plasmatic level of creatinine of >1.4 mg/dl was empirically established as a threshold for possible renal failure, even in the earliest stages, showing 14 (19.4%) patients with renal insufficiency according to this criterion. 

All the patients were administered linezolid exclusively by intravenous administration in 31 (43.1%), exclusively orally in 20 (27.8%), and by both routes sequentially in 21 (29.2%). The existence of the antibiotic with oral bioavailability of nearly 100% facilitates sequential therapy: a) once oral tolerance begins; b) if it is utilized since treatment commenced; c) to occasionally continue treatment at home. The mean treatment duration in this study was 16.2±17.5 days, which is slightly longer than that recommended (10-14 days), and it even exceeded the maximum duration recommended in some cases. 

In the present study, DRP were identified in 36 patients (50.0%). In most cases, there were related to indications (15 patients, 20.8%). The causes included in this category correspond to inappropriate prescription, therapeutic duplication and indication without an antibiogram. In other cases, DRP were related to safety (5 patients, 6.9%). In 16 patients (22.2%), problems combining indications and safety were detected. However, no DRP were recorded, which modified antibiotic efficacy. This last category, however, should be considered with caution as directly monitoring patients’ clinical evolution is not always possible. 

These DRP were all significantly higher in the patients treated with linezolid by an AIS (63.6%) than in those treated by an UIS (28.6%). Hence, new studies into extending linezolid indications may be necessary.

This observational study presents some limitations, mainly the smaller number of patients included, the variety of the choice of alternative antibiotics and the treatment duration of the patients making up the sample. However, one of the determinant factors to obtain maximum clinical efficacy is the *in-vitro* determination of the sensitivity levels of the antibacterial activity through minimum inhibiting concentrations (MIC), which mark the concentrations needed to inhibit bacterial growth. Thus, sensitive microorganisms to linezolid present an MIC of ≤2mg/dl^19^. This may be another study limitation as MIC determinations in antibiograms do not form part of our hospital´s protocol. 
